# Haploidentical related donor compared to HLA-identical donor transplantation for chemosensitive Hodgkin lymphoma patients

**DOI:** 10.1186/s12885-020-07602-w

**Published:** 2020-11-24

**Authors:** Luca Castagna, Alessandro Busca, Stefania Bramanti, Maria Raiola Anna, Michele Malagola, Fabio Ciceri, William Arcese, Daniele Vallisa, Francesca Patriarca, Giorgina Specchia, Roberto Raimondi, Raynier Devillier, Sabine Furst, Laura Giordano, Barbara Sarina, Jacopo Mariotti, Attilio Olivieri, Reda Bouabdallah, Carmelo Carlo-Stella, Alessandro Rambaldi, Armando Santoro, Paolo Corradini, Andrea Bacigalupo, Francesca Bonifazi, Didier Blaise

**Affiliations:** 1 Humanitas Cancer Center, BMT Unit, Humanitas Research Hospital, IRCCS, Via Manzoni 56, Rozzano, MI Italy; 2 Hematology Department Azienda ospedaliera Universitaria S Giovanni Battista, Torino, Italy; 3 Division of Hematology and Hematopoietic Stem Cell Transplantation Unit, Ospedale Policlinico San Martino-IRCCS per l’Oncologia, Genoa, Italy; 4 Department of Clinical and Experimental Sciences, Unit of Blood Diseases and Stem Cells Transplantation, ASST Spedali Civili of Brescia, University of Brescia, Brescia, Italy; 5 Hematology and BMT Unit, Ospedale S Raffaele, Milan, Italy; 6 Department of Hematology, Stem Cell Transplant Unit, Rome Transplant Network, “Tor Vergata” University Hospital, Rome, Italy; 7 Hematology Department, Ospedale Gda Saliceto di Piacenza, Piacenza, Italy; 8 Hematology, Department of Medical Area, University of Udine, Udine, Italy; 9 Hematology Section, DAP University of Bari, Bari, Italy; 10 Hematology Department, S. Bortolo Hospital, Vicenza, Italy; 11 Hematology Department, Transplantation and Cellular Therapy Unit, Institut Paoli-Calmettes, Marseille, France; 12 Humanitas Cancer Center, Statistical Unit, Humanitas Research Hospital, Rozzano, Italy; 13 Department of Hematology, Medical School, University of Ancona, Ancona, Italy; 14 Hematology Department, Lymphoma Program, Institut Paoli-Calmettes, Marseille, France; 15 Hematology and Bone Marrow Transplant Unit, Azienda Ospedaliera Papa Giovanni XXIII, Bergamo, Italy; 16 Hematology and Bone Marrow Transplant Unit, Fondazione IRCCS Istituto Nazionale Tumori and University of Milano, Milan, Italy; 17 Istituto di Ematologia, Università Cattolica del Sacro Cuore, Fondazione Policlinico Universitario Gemelli, Rome, Italy; 18 Institute of Hematology and Medical Oncology, L and A Seràgnoli, St Orsola-Malpighi Hospital, University of Bologna, Bologna, Italy

**Keywords:** Haploidentical transplantation, Hodgkin lymphoma, Reduced intensity conditioning regimen

## Abstract

**Background:**

Allogeneic stem cell transplantation from haploidentical donor using an unmanipulated graft and post-transplantation cyclophosphamide (PT-Cy) is growing. Haploidentical transplantation with PT-Cy showed a major activity in Hodgkin lymphoma (HL), reducing the relapse incidence. The most important predictive factor of survival and toxicity was disease status before transplantation, which was better in patients with well controlled disease.

**Methods:**

We included 198 HL in complete (CR) or partial remission (PR) before transplantation. Sixty-five patients were transplanted from haploidentical donor and 133 from a HLA identical donor (both sibling and unrelated donors). Survival analysis was defined according to the EBMT criteria. Survival curves were generated by using Kaplan-Meier method and differences between groups were compared by the log rank test or by the log rank test for trend when appropriated.

**Results:**

The PFS, OS, and RI were significantly better in patients in CR compared to PR (55% vs 29% *p* = 0.001, 74% vs 55% *p* = 0.03, 27% vs 55% *p* <  0.001, respectively). The 2-year PFS was significantly better for HAPLO than HLA-id (63% vs 37%, *p* = 0.03), without difference in OS. The 1-year NRM was not different. The 2-year relapse incidence (RI) was lower in the HAPLO group (24% vs 44%, *p* = 0.008). Patients in CR receiving haplo HSCT showed higher 2-year PFS and lower 2-year RI than those allografted with HLA-id donor (75% vs 47%, *p* <  0.001 and 11% vs 34%, *p* < 0.001, respectively). In multivariate analysis, donor type and disease status before transplantation were independent predictors of PFS as well as they predict the risk of relapse. Disease status at transplantation and age were independently associated to OS.

**Conclusions:**

Nonetheless this is a retrospective study, limiting the wide applicability of results, data from this analysis suggest that HLA mismatch can induce a strong graft versus lymphoma effect leading to an enhanced PFS.

## Background

Allogeneic hematopoietic stem cell transplantation (allo-HSCT) represents a potential curative option for patients with relapsed or refractory Hodgkin’s lymphoma (HL) [1–5], and has shown to be offer a survival advantage, over chemotherapy alone, for patients relapsing after an autologous transplant [4]. However, an HLA-identical related or unrelated donor is available only for a subset of patients, and family mismatched donors are warranted for such patients. The Baltimore group has shown that T cell-replete haploidentical transplantation, is feasible following a nonmyeloablative conditioning regimen (NMAC) and post-transplant cyclophosphamide (PT-Cy), with a good toxicity profile [6]. In a retrospective study on patients with HL, the relapse risk was decreased in patients grafted from a haploidentical (HAPLO) donor, compared patients grafted from HLA identical sibling (SIBS) or unrelated donors (UD), with a 2-year progression-free survival (PFS), of 51% for HAPLO versus 23% for SIBS and 29% for UD donors [7]. These results suggested a peculiar immunological graft-versus tumor effect of HAPLO donors against HL cells [8–10].

Recently, lymphoma patients grafted with HAPLO donors (using the Baltimore platform) was compared with patients grafted with SIBS or matched unrelated donor (MUD), in 3 registry based studies [11–13]. These studies showed comparable outcome, with a lower incidence of chronic GVHD after HAPLO grafts. The EBMT study, focusing on HL, also showed comparable outcome, but included chemosensitive and chemorefractory patients. We hypothesized that chemosensitive HL patients would be best suited to test whether HAPLO donors would induce a stronger graft versus lymphoma effect, as compared to SIBS and UD. We are now reporting a comparative analysis of 198 patients with HL with chemosensitive disease receiving allo-HSCT from SIBS/MUD, or HAPLO donor.

## Methods

In this analysis we included HL patients receiving haplo-HSCT, in 3 Centers (Humanitas Research Hospital, Rozzano; Ospedale San Martino, Genova; Institut Paoli Calmettes, Marseille, France) and HL patients receiving, during the same period (from 2009 to 2012), a HLA identical transplantation, both from related or unrelated donor, selected by centers from the Gruppo Italiano Trapianto di Midollo e cellule staminali periferiche (GITMO). This retrospective study was approved by ethical committee (ONC/OSS 04/2015).

Search for allogeneic stem cell donor was initiated for patients relapsing after high-dose chemotherapy or requiring three or more chemotherapy lines to control initial disease, or in case of disease refractory to salvage chemotherapy. For patients lacking an HLA-id sib or UD, haploidentical donor was searched.

We analyzed if the survival was different between HLAid and MUD, and the 2-year PFS and OS were not statistically different (HLAid vs MUD, 2-y PFS 28 vs 38% (p 0.47); 2-y OS 73% vs 53% (p 0.087), allowing us to combine them.

### Inclusion criteria

We included only chemosensitive (complete remission, CR or partial remission, PR) patients before allo-HSCT, performed during the same time frame (from 2010 and 2014): 65 patients received haploidentical transplantation (Haplo-HSCT) and 133 HLA-identical (HLA-id) transplantation, both from sibling and unrelated donors. The institutional review board of each center approved the study.

### Haploidentical transplantation

Patients were conditioned using nonmyeloblative conditioning regimen including Cy 14.5 mg/kg on days − 5 and − 6, fludarabine 30 mg/m^2^ from day − 6 to day − 2 and low-dose TBI (2 Gy) on day − 1. RIC regimen was used in one patient associating consisting of thiotepa 10 mg/kg, fludarabine 30 mg/m^2^, and cyclophosphamide 30 mg/kg. GVHD prophylaxis consisted of Cy 50 mg/kg administered on days + 3 and + 4. On day + 5, tacrolimus or cyclosporine A (CyA) and mycophenolate mofetil (MMF) were started. Tacrolimus (FK 506, at a total dose of 1 mg) was administered as a continuous infusion until discharge and was converted to an oral formulation thereafter. The doses were adjusted to obtain serum levels between 10 and 20 ng/ml. CyA was dosed at 3 mg/kg as a continuous infusion until discharge and was converted to an oral formulation thereafter. The CyA doses were modified to obtain serum levels between 100 and 200 ng/ml. MMF was administered at 15 mg/kg po three times per day until day + 35. G-CSF was started on day + 5 in all the patients.

### HLA identical transplantation

We will consider together patients grafted from identical siblings, and patients grafted from 8/8 matched unrelated donors, and we will refer to these as HLA identical transplants. Several reduced conditioning regimens were used for patients grafted from HLA identical donors, mostly including thiotepa. GVHD prophylaxis consisted of CyA and methotrexate (day + 1, + 3, + 6, +/− 11), combined with Thymoglobuline for UD transplants.

### Stem cell sources and donors

In case of haploidentical transplantation, potential donors were typed at the HLA-A, HLA-B, HLA-C, HLA-DRB1, and HLA-DQB1 at a high resolution level. All the donor/recipient pairs exhibited a median of 4 mismatches (range 2–5) on the unshared haplotype. The donors underwent bone marrow harvest under general anesthesia, and the target dose was 4 × 10^8^ nuclear cells/kg of recipient weight. In Marseille, 20 donors underwent BM harvest, while 23 were mobilized by the subcutaneous administration of G-CSF (5 to 6 days at 10 μg/kg/day). The target yield was 4 × 10^6^ CD34/kg. Unmanipulated stem cells were infused on day 0.

### Supportive care

Each center applied specific supportive care in terms of antimicrobial prophylaxis and transfusion policies.

### Statistical analysis

The aims of this study were to evaluate the impact of donor and disease status on outcome of allo-HSCT in HL patients.

Patients were compared with respect to the main clinical pathological characteristics: gender, age, number of previous lines of therapy, therapeutic program, disease status at transplant, CMV, ATG and conditioning regimens. Differences between groups were evaluated by using the T-test for continuous distributions and the Chi-square or the Fischer exact test for categorical distributions.

Survival analysis was defined according to the EBMT criteria. Progression free survival (PFS), relapse incidence (RI), overall survival (OS) and non-relapse mortality (NRM) were evaluated starting time from allogeneic transplantation. Cumulative incidence was considered to estimate aGVHD, cGVHD, relapse incidence (RI) and non-relapse mortality (NRM) after haploidentical transplantation. Survival curves were generated by using Kaplan-Meier method and differences between groups were compared by the log rank test or by the log rank test for trend when appropriated. To test whether the differences in cumulative incidence were statistically significant the Gray’s method was applied. All analysis were performed using Stata 13 and R 3.0.3 softwares.

## Results

Patient and transplantation characteristics are reported in Table 1. The median time of observation for surviving patients was 29 months (range 14.1–57.4), with no difference between haplo and HLAid.

**Table 1:** Patient and transplantation characteristics

	All pts. *N* = 198	HLAid *N* = 133	Haplo *N* = 65	*p*
Age, years (median, range)	32 (18–66)	32 (18–65)	31 (18–65)	0.8
Sex M/F	113/85	77/56	36/29	0.7
Number of CT lines (median, range)	2 (2–12)	2 (2–9)	4 (2–12)	< 0.001
Previous HDC	170/198 (86%)	110/133 (83%)	60/65 (92%)	0.007
Disease status at transplantation				
CR	119 (60%)	82 (62%)	37 (60%)	
PR	79 (40%)	51 (38%)	28 (40%)	
Donors				NA
HLA sibling	/	57 (43%)	/	
MUD	/	76 (57%)	/	
Haplo	/	/	65	
Stem cell source				
PBSC	134 (60%)	114 (83%)	20 (31%)	< 0.001
BM	63 (31%)	18 (13%)	45 (69%)	
miss	1 (9%)	1 (4%)	/	
ATG prophylaxis GVHD				NA
No	122 (62%)	57 (43%)	/	
Yes	76 (38%)	76 (57%)	/	
Conditioning regimens				
NMAC	58 (29%)	2 (2%)	58 (89%)	< 0.001
RIC	101 (51%)	92 (69%)	7 (11%)	
MAC	39 (20%)	39 (29%)	/	

*CR* complete remission, *PR* partial remission, *MUD* matched unrelated donor, *BM* bone marrow, *PBSC* peripheral blood stem cells, *HDC* high-dose chemotherapy

### Engraftment

In the HAPLO group, the median time to reach an ANC of more than 0.5 × 10^9/L, was 20 days (range 14–32), and in the HLAid group 14 days (range 7–47). Failure to engraft was seen in 3 HAPLO patients (7%) and in 2 patients (6%) in the HLAid group. In the haplo group, 2 out 3 patients had graft failure because of donor specific antibodies (DSA), and 1 patient died of pneumonia before engraftment.

### Acute and chronic GVHD

In the HAPLO and HLA-id group, the incidence of grade 2–4 acute GVHD was 15% vs 16% (*p* = 0.9) and chronic GVHD were 18% vs 32% (*p* = 0.06), respectively.

### Survival, relapse and NRM (Table 2)

**Fig. 1:**
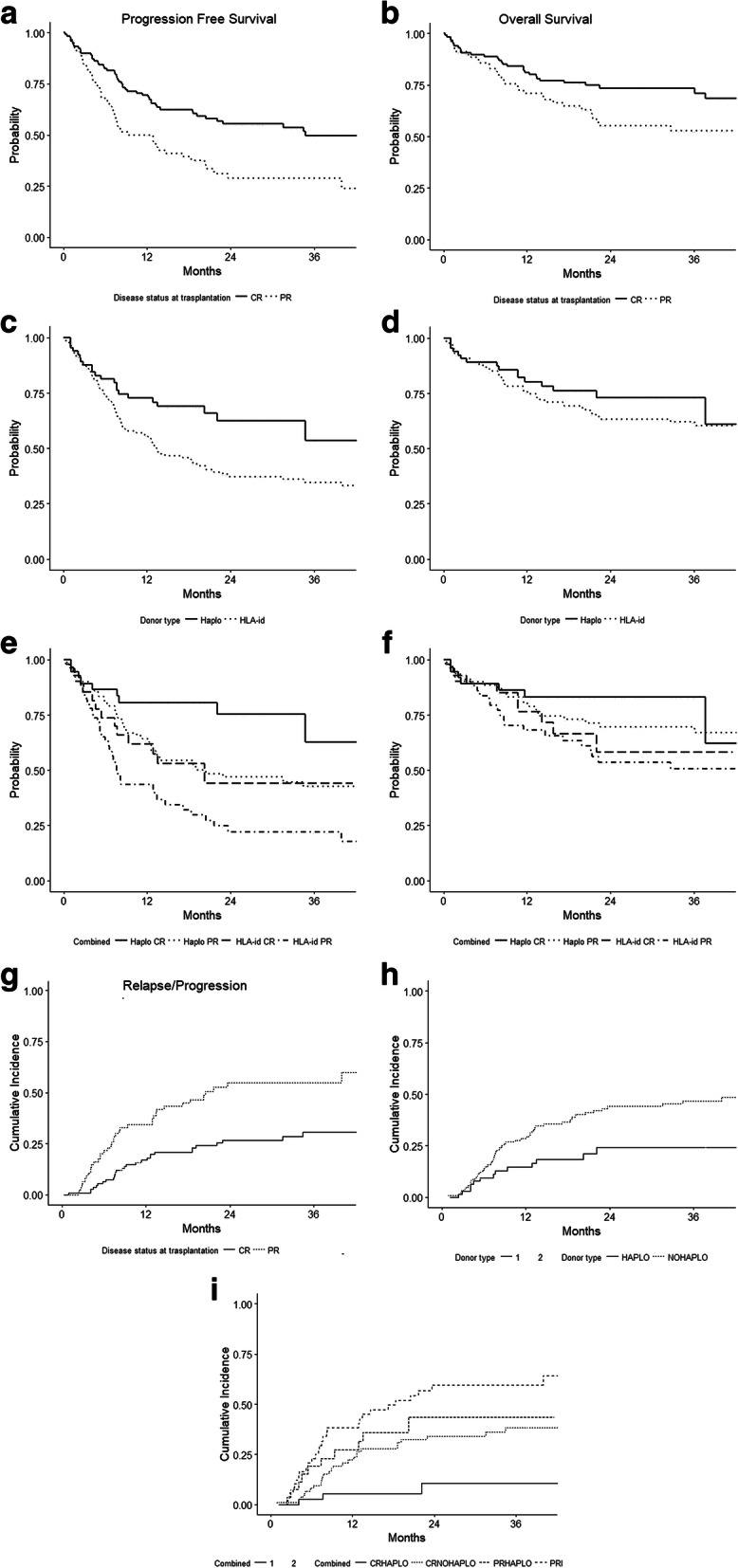
PFS by disease status (**a**), donor type (**c**), and combining disease status and donor type (**e**), OS by disease status (**b**), donor type (**d**), and combining disease status and donor type (**g**)

**Fig. 2:**
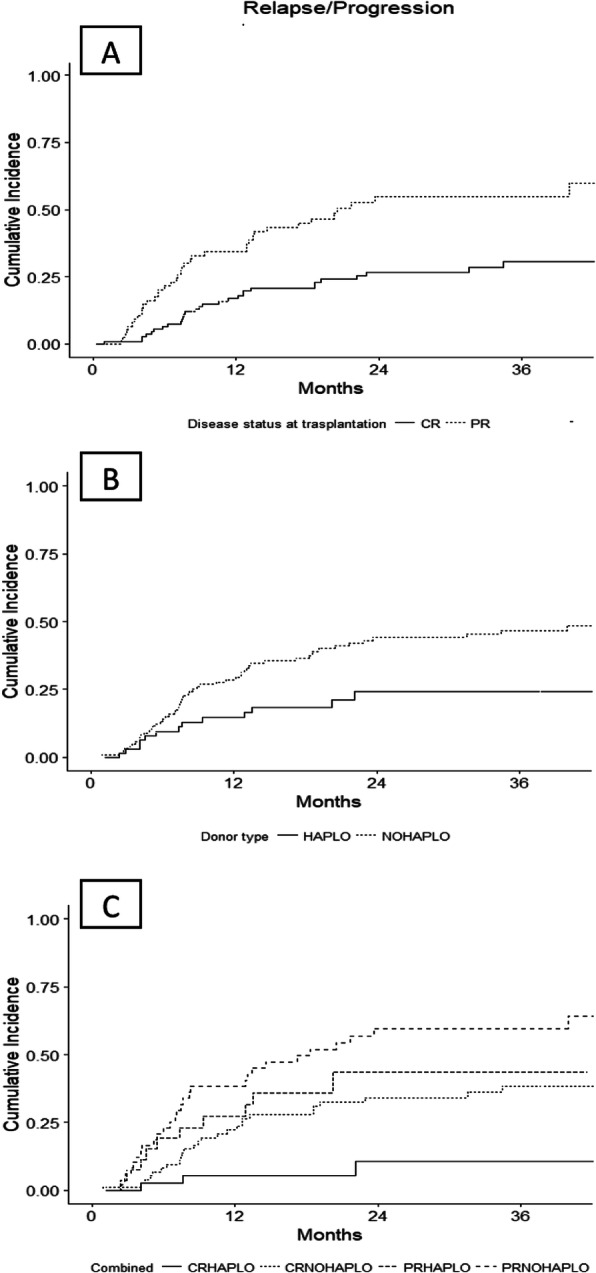
Relapse incidence by disease status (**a**), donor type (**b**), and both (**c**)

The overall 2-year PFS, OS, relapse incidence (RI), and 1y-NRM were 45, 66, 38, and 14%, respectively. The PFS, OS, and RI were significantly better in patients in CR compared to those in PR (55% vs 29% *p* = 0.001, 74% vs 55% *p* = 0.03, 27% vs 55% *p* < 0.001, respectively. (Figs. 1a, b, 2a), whereas the 1-year NRM was similar (15% vs 16% *p* = 0.9).

**Table 2:** Main outcomes in all patients and specific sub-groups

	N	2y PFS	***p***	2y OS	***p***	2y RI	***p***	1y NRM	***p***
**All**	18	45%		66%		38%		14%	
**CR vs PR**	119 vs 79	55% vs 29%	0.001	74% vs 55%	0.03	27% vs 55%	< 0.001	13% vs 16%	0.8
**Haplo vs HLAid**	65 vs 133	63% vs 37%	0.03	67% vs 63%	0.6	24% vs 44%	0.008	13% vs 15%	0.9
**CR Haplo vs**	37	75%	< 0.001^a^	83%	0.1	6%	<	14%	0.8
**CR HLAid vs**	82	47%		67%		34%	0.001^a^	13%	
**PR Haplo vs**	28	44%		58%		44%		11%	
**PR HLAid**	51	22%		54%		60%		18%	

*CR* complete remission, *PR* partial remission, *PFS* progression free survival, *OS* overall survival, *RI* relapse incidence

^a^trend

The 2-year PFS for HAPLO and HLA-id HSCT, was 63% vs 37% (*p* = 0.03) (Fig. 1c), the 2-year OS 67% vs 63% (*p* = 0.6) (Fig. 1d), and the 1-year NRM was 13% vs 15%, (*p* = 0.9), respectively. The 2-year RI was significantly lower in the HAPLO group (24% vs 44%, *p* = 0.008) (Fig. 2b).

We analyzed the clinical outcome in specific sub-groups of patients, combining disease status and donor. As shown in Table 2, patients in CR receiving a haplo HSCT showed a significantly better 2-year PFS (Fig. 1e) and lower 2-year RI (Fig. 2c) compared to those allografted with HLA-id donor (75% vs 47%, *p* < 0.001 and 11% vs 34%, *p* < 0.001, respectively). No statistically significant differences were founded in terms of OS (Fig. 1g) and NRM. In PR patients, we found a similar advantage for HAPLO donors with a 2-year PFS of 44% vs 22%, *p* < 0.001 (Fig. 1e); and the 2-year RI was 44% vs 60%, *p* < 0.001 (Fig. 2c). Finally, patients in PR receiving transplantation from a HLAid donor showed the worst outcome in terms of PFS and RI (22 and 60%, respectively) (Figs. 1e and 2c).

### Univariate and multivariate analysis (Table 3)

In univariate analysis, age (with a cut off at 45 years), recipient CMV serostatus, ATG, conditioning regimen, therapeutic program (relapse post-HDC), and disease status (CR vs PR) did not affect survival. Donor type (HAPLO vs HLA-id) influenced the PFS (*p* = 0.06), but not the OS. In multivariate analysis, donor type (haplo vs HLA-id HR 0.51, *p* < 0.001) and disease status before transplantation (CR vs PR HR 0.37, *p* = 0.014) were independent predictors of PFS as well as they predict the risk of relapse (donor type HR 0.37, *p* < 0.001; disease status, HR 0.43, *p* = 0.006). Disease status at transplantation and age (as continuous variable) were independently associated to OS (CR vs PR HR 0.57, *p* = 0.02; age HR 0.04, *p* = 0.006).

**Table 3:** Multivariate analysis

	HR (CI95%)	***P*** value
**PFS**		
Disease status (CR vs PR)	0.51 (0.34–0.75)	< 0.001
Haplo vs HLAid	0.56 (0.35–0.89)	0.014
**OS**		
Disease status (CR vs PR)	0.57 (0.35–0.93)	0.023
Age (continuous variable)	0.04 (1.00–1.05)	0.043
**Relapse**		
Haplo vs HLAid	0.37 (0.23–0.60)	< 0.001
Disease status (CR vs PR)	0.43 (0.43–0.79)	0.006

*CR* complete remission, *PR* partial remission, *PFS* progression free survival, *OS* overall survival

## Discussion

In this study, we have tested the effect of donor type on outcome, in chemosensitive HL patients, undergoing an allogeneic transplant from haploidentical donor, using a T-replete stem cell source and PT-Cy, or HLA identical donors, which included matched siblings and matched unrelated donors.

We have shown that, haplo-HSCT result in a significantly superior 2-year PFS (63%), when compared to HLA identical transplants (37%). This is due to a significantly reduced risk of relapse in HAPLO patients: the 2-year cumulative incidence of relapse was indeed 24% for HAPLO and 44% for HLA identical grafts. This was seen despite the fact that HAPLO patients were all prepared with a non myeloablative, extremely mild conditioning regimen, whereas a proportion of patients in the HLA identical cohort received a more intensive regimen, recently shown to have a role in reducing tumor burden in HL [14].

Remission status remained significant prognostic variable in univariate and multivariate analysis, with CR patients having superior outcome as compared to partial remission patients, in keeping with data in the literature. When combining donor type and disease status, 37 HAPLO grafts in CR had the best 2 year PFS (75%), with the lowest risk of relapse (6%). HLA identical grafts in CR patients (*n* = 82) and HAPLO grafts in PR (*n* = 28) had quite comparable outcome, with a PFS of 47 and 44%, and a risk of relapse of 34 and 44% respectively. The worst outcome was in 51 patients in PR grafted from HLA identical donors (22% PFS and 60% relapse). This was confirmed in a multivariate analysis on PFS and relapse, showing that donor type and remission status were independent predictors.

Overall survival was predicted only by disease status and patients age, but not by donor type: this is due to the fact that non relapse mortality was similar in patients grafted from HAPLO and HLA identical donors, whereas is was influenced by disease status and age, In addition post-transplant relapse can be rescued in HL patients [15, 16], and therefore a strong effect on relapse does not necessarily translate on survival, at least in the short/medium term.

Another important point was the toxicity of haploidentical transplantation: both in CR and PR groups, the 1-year NRM was low and not different as summarized in Table 2. Similarly, the incidence of grade 2–4 aGVHD was similar, despite the fact that 31% of HAPLO grafts were performed using peripheral stem cells. Chronic GVHD in the haplo group was in line with previous reports, and basically lower compared to that observed in HLAid group.

These results challenge those reported in other comparative studies from CIBMTR, including Hodgkin and non-Hodgkin lymphomas, and one study from the EBMT on HD patients only. The CIBMTR study compared 44 patients grafted from haploidentical donors, to 178 patients transplanted from a matched related donor [11]. They found no difference in terms of 3-year PFS and RI (48% vs 48 and 37% vs 40%, respectively). In the second CIBMTR study, haploidentical transplants were compared to patients grafted from matched unrelated donor. Again, no differences were observed in terms of PFS and RI [12]. The recent EBMT study included HL patients only and again the survival, NRM and aGVHD incidence were similar between haplo-HSCT, matched related donor and matched unrelated donor [13]. Other studies from French Society of transplantation showed similar results [14]. The question is why our present study would instead show an advantage for HL patients receiving a HAPLO graft: one possibility is the inclusion criteria, since we enrolled exclusively patient with chemosensitive disease, whereas in the previous studies the percentage of refractory patients was consistent (ranging from 5 to 30%) [11–14]. Relapse rates are very high in chemoresistant patients, also after an allogeneic transplant, and this may quench the beneficial effect of given donor type.

But why should HAPLO grafts have a stronger graft versus HL effect? We hypothesize two possibilities. In the first place donor lymphocytes may interact more effectively with Hodgkin’s cellular microenvironment, inducing apoptosis, and indirectly affecting survival of Reed Sternberg cells [17]. The second possibility comes from a recent study on check-point inhibitor activity against HL: in this study, the activity of check-point inhibitors was higher when HL cells expressed HLA class II molecules [18]. We speculate that class II HLA mismatches in a HAPLO transplant, may enhance the anti-tumoral effect of donor CD4+ T cells.

The present study has several and important limitations, mainly due to its retrospective nature, such that we cannot exclude some bias in patient’s selection. The use of haploidentical transplantation started in 2010, therefore patients with an indication of HSCT and without a HLA-id donor before that date did not undergo an allogeneic HSCT. In addition, GvHD prophylaxis was different, based on PT-CY in the HAPLO patients and on ATG in the HLA identical grafts, although in a previous analysis, ATG did not significantly influence the outcome in lymphoma patients receiving allogeneic stem cell transplantation [10]. Finally, the conditioning regimen was the same for all HAPLO patients and rather heterogeneous in the HLA identical group, although there is little evidence in the literature that conditioning regimens influence the outcome of allogeneic HSCT in HL patients [19].

## Conclusions

This study suggests that HLA haploidentical transplantation, using T-cell replete stem cells and PT-Cy, is more effective than HLA identical grafts in chemosensitive advanced HL, due to greater antitumor activity, challenging the question of donor choice. A prospective comparative study is needed, possibly using the same conditioning regimen and the same prophylaxis for graft versus host disease, in order to assess the role of HLA haploidentical grafts for patients with Hodgkin lymphoma.

## Data Availability

The datasets used and/or analyzed during the current study are available from the corresponding author on reasonable request.

## Abbreviations

HL: Hodgkin lymphoma
CR: Complete remission
PR: Partial remission
PFS: Progression free survival
OS: Overall survival
RI: Relapse incidence
NRM: No relapse mortality
allo-HSCT: Allogeneic hematopoietic stem cell transplantation
NMAC: Nonmyeloablative conditioning regimen
PT-Cy: Post-transplant cyclophosphamide
SIBS: HLA identical sibling
UD: Unrelated donor
HAPLO: Haploidentical
CyA: Cyclosporine A
MMF: Mycophenolate mofetil
FK 506: Tacrolimus
GVHD: Graft versus host disease
